# The Treatment of Epilepsy

**Published:** 1892-08

**Authors:** Frederick Peterson

**Affiliations:** New York; Formerly Professor of Pathology in the University of Buffalo; Attending Physician New York Hospital for Nervous and Epileptic; Chief of Clinic, Nervous Department, Vanderbilt Clinic, College of Physicians and Surgeons; 201 West Fifty-fourth Street


### THE TREATMENT OF EPILEPSY.

By FREDERICK PETERSON, M. D., New York.

Formerly Professor of Pathology in the University of Buffalo; Attending Physician New York Hospital for Nervous and Epileptic; Chief of Clinic, Nervous Department, Vanderbilt Clinic, College of Physicians and Surgeons.

THERE has been great advance of late years in the treatment of the epileptic, and I am not aware that there has anywhere appeared a condensed, practical account of the methods in vogue at the present time. Somehow or other, the epileptic and his needs as an individual have been forgotten, although empirical attempts enough to cure his obstinate malady have been made for many centuries.

In the first place, we have come latterly to look upon epilepsy not as a disease *sui generis*, but as a symptom of a number of pathological conditions, some in the central nervous system, some in the blood, and some of a reflex nature. At any rate, each and every case must be studied upon its own merits, and in the light of modern discoveries in the nervous system. It is only thus that we can learn which cases are particularly well fitted for dietetic and medicinal treatment, and which require the intervention of the surgeon. It is not the province of this article to point out the methods of diagnosis. With due care and continuous observation the physician will differentiate the features of attacks, whether petit or grand mal, nocturnal or diurnal, or a larval form. He will look for gastro-intestinal irritation and ocular disorders; he will examine the genital organs; he will exclude hysteria; he will make a chemical study of the urine for the light it may throw upon nutritional disturbances. Then the character of the seizure, its mode of origin, whether preceded by sensory hallucinations (auræ), and its limitations to certain groups of muscles, or its general nature must be studied. After such careful investigation a line of treatment may be marked out.

*Surgical Treatment.*—A limited number of epileptics may be treated surgically with benefit in the vast majority of cases, and with good hope of cure in a select few. The cases eligible for trephination are those that give good evidence of a cortical lesion, such as a palpable depression in the skull with a history of an injury, or where the epilepsy is distinctly Jacksonian in character, thus indicating a certain point of cortical irritation, where the epileptic explosion begins. Trephining, when done by a skilful surgeon, and under strictest antiseptic precautions, may be looked upon as a comparatively safe operation. It should be resorted to more often than it is as a preventive measure. I refer to cases of head injury which seem, on first examination, to be trivial, but which, in the course of time, develop epilepsy. A case in point presented himself at the Vanderbilt Clinic a few days ago:

A boy of twenty was struck on the head by a bottle falling from a third story window. There was no loss of consciousness, and apparently no injury to the skull. At any rate, the scalp wound, which was severe, was sewed up and healed readily. This was three years ago. During the first year after the accident he had one epileptic fit ; during the second year, two or three ; now he has them two or three times weekly. There is a large scar and an apparent depression over the right fronto-parietal region, very near the median line.

I consider this case a proper one for trephination, although there are no focal symptoms of the epilepsy. At this point will be found either a depressed spiculum of bone, a meningeal thickening, or a cortical sclerosis, requiring removal.

*Dietetic Rules.*—Eating and drinking should be always moderate. Alcohol, coffee, and tea should be eschewed, and smoking also, in most cases, forbidden. Undoubtedly the best food for the epileptic is a vegetable and milk diet, but meat should not be altogether excluded. A vegetable diet does no harm to an adult, but should be combined with fats in the shape of oils, butter, or fat meat. We know very well that whole nations live almost exclusively upon such a diet, as I have myself observed among the hard-working, muscular Egyptian fellaheen. But growing children require proportionately more food than adults. They have not only the daily waste to supply, but a growing organism to build up, and to form a large amount of proteid (Bunge) ; and a mixed though light and easily digested diet should be ordered. The dinner should be in the middle of the day. The evening meal should be light. There is as yet no explanation of the nature of the epileptic explosion in the convulsive centers. Theoretically, they are looked upon as nutritional disturbances in nitrogenous compounds in nerve cells. That we have to deal with profound chemical changes in the central nervous system in most of these cases, there is no doubt. Recent studies in the urine of epileptics have demonstrated the truth of this ; and after a time we shall, by more careful study, be able to modify by diet alone the frequency of seizures in many cases.

*Employment.*— It is too frequently the fact that the unfortunate epileptic is given a prescription, a few words upon diet, and then allowed to take his own course. But the employment of mind and body is of vital importance in most of these cases. An out-of-door life in the country is that best adapted for them. If the patients are so badly off that they cannot fulfil their usual social obligations, get an education in the schools, or carry on their trades or professions among their fellows, they must be sent to an institution for epileptics, where provision is made for all such needs. The best institution in the world of this kind is that at Bielefeld, Germany, which I have fully described on many occasions,[1] and whither I have had the pleasure of sending several patients from various states in this country, to the great happiness of themselves and the relief of their families. I am glad to say that similar institutions are now being organized in this country. There is one already in embryo at Santa Clara, California; one nearly completed at Gallipolis, Ohio, and New York will have located a site and secured plans for buildings on the colony system before the end of this year.

*Hydro-therapy.*—Cold shower-baths and cold sponge-baths, daily, are beneficial. The shower-baths should be rain-like in character, that is, not too forcible.

In many cases a morning and evening bath (the "half-bath") proves very serviceable. The "half-bath" is taken in a bath-tub only half-filled with water, and when taken should be accompanied by energetic rubbing of the patient by an attendant. This bath lasts five minutes, and the temperature should be not under fifty and not over seventy degrees Fahrenheit.

Where there is evidence of hyperemia and increased blood pressure in the head, the cold cap is useful.

While these are the general indications for hydro-therapy, certain measures are often of use at the time of seizures. During a fit or during a *status epilepticus* it will be observed that there is one of two vascular conditions present: either the face is pale and there are signs of brain anemia, and in this case warm wet compresses should be applied to the head and genitals, accompanied by friction of the trunk upward, the body being placed with head low and arms uplifted; or there is turgescence of vessels in the head, the face is red, the carotids beat strongly, and under such conditions a contrary procedure is indicated—cold compresses to the

1. *N. Y. Medical Record*, April 23, 1887; Journal of Nervous and Mental Diseases, December, 1889; *State Charities Record*, N. Y., June, 1890; Journal Nervous and Mental Diseases, July, 1892.

head, neck and genitals, strong, wet beating of the feet, with a high position of the head.

*Treatment with Drugs.*— For the great majority of cases of grand mal and petit mal, a bromide will be found by far the most useful drug. Only one bromide should be used, and the experience of some of the best practitioners (like Gowers), and of one of the greatest epileptic colonies for twenty-five years (Bielefeld), is that the bromide of potassium is the best salt, having the least irritating effect upon the gastric mucosa. It is a pity that it cannot always be obtained in pure form, for, as ordinarily sold, it has impurities, like chlorate of potash, to the extent of six per cent. I hope some chemist will undertake a careful comparative analysis of the various bromides in our market, as it is very important that a pure drug be employed. The bromide of potash may be prescribed in powders of ten to fifteen grains each, to be taken with plenty of water after meals, or may be given in a solution in water simply, as follows :

℞.—Potassii bromidi .............40 grammes (ʒ x.)

Aquæ puræ ................200 grammes (ʒ l. ) M.

Which is a proportion of one-fifth of the drug to a teaspoonful, making a dose of twelve to fifteen grains, according to the size of the teaspoon. Or it may be given in a one to four solution :

℞.—Potassii bromidi .............50 grammes (℥ i.)
Aquæ puræ ................200 grammes (℥ iv.) M.

While, in the ordinary diurnal forms, it is, perhaps, as well to give this quantity in three doses daily, it is, as a rule, better in the nocturnal form to prescribe either an extra dose at night or the whole amount of forty to sixty grains in one dose at bedtime, in a full glass of water.

While children will bear large doses of bromide fairly well, it is better in those from ten to sixteen years of age to begin with ten grains, three times daily, and under ten years, still less.

Each week an additional dose should be added, for three or four weeks, the object being to push the drug to the limit of physiological tolerance — very nearly to the state known as bromism. But extreme discretion must be used in the early part of the treatment, to discover the patient's susceptibility to the remedy, and to guard against its harmful effects.

Chloral may often be advantageously combined with the bromide (Seguin), particularly in cases with a tendency to severe cutaneous eruptions, and when thus administered, the bromide itself should be proportionately reduced.

Where indicated, other agents may be employed for concomitant conditions,—iron for anemia, arsenic for the acne, and saline waters and salts for constipation.

To sum up in a brief sentence the treatment for epilepsy in general of all forms, must consist of careful regulation of the diet, hydro-therapeutic measures, out-of-door employment, and the bromide of potash.

Occasionally, there are cases that do not do well on a bromide. To these may be given borax in fifteen to twenty grain doses three times daily after eating, or lactate of zinc, seven to twelve grains at a dose. Tincture of simulo will, at times, be found useful in one to two drachm doses (Starr).

In petit mal I have seen excellent results from the use of nitroglycerin in doses of one-one-hundredth of a grain three times daily.

In the nocturnal form, tincture of belladonna, five to ten minims, or atropine, one-one-hundred-and-twentieth grain alone is, at times, strikingly effective.

For patients who feel their attacks coming on, we are in the habit, at the Vanderbilt Clinic, of providing a wide-mouthed, glass-stoppered bottle containing nitrite of amyl in a plug of cotton. When an attack is impending, the patient smells of the vapor, and often in this way wards off a seizure or diminishes its severity.

Whenever a case is taken in charge, he should be provided with a blank form, upon which not only should the patient register his attacks as they occur, but the physician should write his regulations for baths and diet, and put down in cipher, for his own use, his treatment; the blank to be returned to the doctor in charge at the end of a year. I have had made for my own use a special form of blank by G. P. Putnam's Sons, of New York, which is exceedingly serviceable. It is printed on a heavy paper, so that it will not wear out when carried in the pocket for a year; has spaces for monthly notes of treatment; for a daily record of attacks; for a monthly, half-yearly, and annual statement of the number of seizures; for the orders as to baths; and, furthermore, the proper regulations as to diet and kinds of food are printed upon the back of the register. I should be happy to send a sample of this epileptic record to any one who desires it, or application may be made to the publishers.

201 West Fifty-fourth Street.

